# The acoustic startle response in 22q11 deletion syndrome: from animal models to humans

**DOI:** 10.3389/fnins.2025.1630109

**Published:** 2025-08-15

**Authors:** Sidney Imes, David A. Parker, Marissa Chen, Joseph F. Cubells, Elaine F. Walker, Erica Duncan

**Affiliations:** ^1^Atlanta VA Health Care System, Decatur, GA, United States; ^2^Department of Psychiatry and Behavioral Sciences, Emory University School of Medicine, Atlanta, GA, United States; ^3^Department of Psychology, Emory University, Atlanta, GA, United States; ^4^Department of Human Genetics, Emory University School of Medicine, Atlanta, GA, United States

**Keywords:** 22q11.2 deletion syndrome, acoustic startle response, psychosis risk, genetic high risk for psychosis, psychophysiology

## Abstract

The startle response is a reflexive contraction of skeletal musculature in response to a strong acoustic stimulus that is evolutionarily preserved across species. There is a broad and comprehensive literature connecting components of the startle response such as latency, magnitude and pre-pulse inhibition, to psychosis status and risk. In this review, we examine the startle response in human subjects with 22q11.2 Deletion Syndrome (22qDel) and in analogous animal models. 22qDel is a copy number variant disorder typically involving ~1.5 to 3 Mb of DNA on the proximal 22q region, which occurs in approximately 1 in 2000–6,000 births, and serves as the most robust single genetic predictor of psychosis risk (conferring ~30x higher risk). By comparing the human literature directly to the genetic mouse models, we identify areas of convergence and divergence between human and animal results and highlight gaps related to differences in neurodevelopmental stages, experimental design, stimulus outcome measurements, and genetic deletion areas in each animal model. We then highlight the translational power of the acoustic startle response and how it can be studied in conjunction with more basic cellular investigations related to basic neural function and responsiveness. Because the acoustic startle response is seen across vertebrate species with well characterized circuitry, we argue for using the acoustic startle response as a translational biological probe of underlying neurobiology relevant to 22qDel and by extension, psychosis and psychosis risk.

## Introduction

22q11.2 deletion syndrome (22qDel) is a chromosomal interstitial-deletion disorder that occurs in 1 in 2000–6,000 live births (Botto et al., 2003; Olsen et al., 2018; Blagojevic et al., 2021). The deletion confers risk for a variety of neurodevelopmental and psychiatric disorders, the most documented of which are psychotic disorders. Twenty to 30 % of individuals with 22qDel develop SCZ by early adulthood, making 22qDel one of the most robust genetic predictors of schizophrenia (SCZ; Jonas et al., 2014; Olsen et al., 2018). SCZ associated with 22qDel is phenotypically comparable to idiopathic SCZ at the level of both symptoms and brain morphology (Bassett et al., 2003, 2005; Sun et al., 2020; Supekar et al., 2024; Zinkstok et al., 2019).

The acoustic startle response (ASR) is a promising translational target for studying the neurobiological pathways that connect the genetic changes in 22qDel with global symptoms in SCZ. The ASR is the reflexive contraction of skeletal musculature that is mediated by a pontine-based, three-synapse subcortical neural circuit in humans, non-human primates, and rodents on exposure to a strong acoustic stimulus (Koch, 1999). It has been studied extensively in SCZ and other psychiatric disorders. Changes in the ASR, such as greater startle latency, associate with psychosis symptoms and can predict conversion to psychosis for clinical high risk groups (Braff et al., 2001; Swerdlow et al., 2008, 2016; Cadenhead et al., 2020).

The 22q11.2 region includes several genes that might affect neuronal processing related to ASR. The PRODH, COMT, DGCR8, TBX1, ZDHHC8, and CXCR4 genes are involved in neuronal development, synaptic function, and dopaminergic, GABAergic and glutamatergic signaling (Boot et al., 2008; Squarcione et al., 2013; de Koning et al., 2015). The PRODH gene affects proline metabolism and the balance between glutamate and GABA synthesis, impacting neuronal activity and sensory processing in 22qDel. The COMT gene, affecting dopamine metabolism, indirectly influences GABAergic function by altering dopamine levels, impacting cognitive functions and sensory processing. These genes may affect ASR through their roles in neurotransmitter signaling within startle circuits, particularly PRODH’s influence on excitatory/inhibitory balance in brainstem nuclei and COMT’s effects on dopaminergic modulation of startle magnitude and habituation. The developmental roles of TBX1 and DGCR8 may also impact the proper formation of the three-synapse pontine circuit underlying the startle response.

As a promising target for translational research, the ASR has been studied in humans with 22qDel and in mouse models of 22qDel. This mini review will summarize and compare findings to date in human subjects with this deletion syndrome and mouse deletion models, and it will identify areas in need of further research.

## ASR

The ASR is a fast, involuntary motor reaction to sudden and intense stimuli, and its core neural architecture is highly conserved across mammalian species (Koch, 1999; Zheng and Schmid, 2023) The response is generated from a simple, three-synapse brainstem circuit: auditory input reaches cochlear root neurons (CRNs), which project directly to giant neurons in the caudal pontine reticular nucleus (PnC), which then activate spinal motor neurons to produce the reflexive motor response (Davis et al., 1982; Lee et al., 1996; Davis, 1997; Koch, 1999; Fendt et al., 2001; Zheng and Schmid, 2023). This circuit enables startle responses within ~10–20 ms of stimulus onset depending on the species. The latency and magnitude of response depend on glutamatergic excitation, particularly via AMPA and NMDA receptors. Blocking these receptors in the PnC has been shown to reduce or abolish ASR in rodent models (Miserendino and Davis, 1993). GluA4 knockout mice that lack a key AMPA receptor subunit essentially lack ASR (García-Hernández and Rubio, 2022). GABA is implicated in prepulse inhibition (Yeomans et al., 2010) but does not directly mediate the ASR.

In humans, the ASR is studied by attaching electrodes to the face to measure the electromyographic signal from the eyeblink response elicited by the startling stimulus (Landis and Hunt, 1939). In rodents, the ASR is studied by movement as captured by a piezoelectric movement-sensitive platform.

There are two ASR variables that have been studied in 22qDel humans and 22qDel mouse models. The first is startle magnitude, which is assessed in humans by measuring the magnitude of the eyeblink response that the auditory stimulus elicits (Graham, 1975). It is measured in rodents via the animal’s movement in response to the stimulus (Hoffman and Searle, 1968; Saito et al., 2020). Human findings on the relationship between startle magnitude and SCZ are mixed; some studies have found reduced magnitude in SCZ vs. control participants (Kumari et al., 2005; Quednow et al., 2006; Minassian et al., 2007; Greenwood et al., 2010; Matsuo et al., 2016), but most studies have found no between-group difference (Braff et al., 1992, 2005; Cadenhead et al., 2000; Parwani et al., 2000; Ludewig et al., 2002; Perry et al., 2002; Wynn et al., 2004; Swerdlow et al., 2006; Takahashi et al., 2008; Hasenkamp et al., 2010; Storozheva et al., 2016).

The second variable is prepulse inhibition (PPI). PPI indicates the reduction in startle magnitude that occurs when the startling sound is preceded by a non-startling tone (Hoffman and Searle, 1968; Graham, 1975). It is used to index sensorimotor gating (San-Martin et al., 2020). PPI impairment has been consistently found in non-22qDel SCZ [Braff et al., 1978, 1992; Hasenkamp et al., 2010; Swerdlow and Light, 2018; Massa et al., 2020; and see reviews in Braff et al., 2001 (human studies of prepulse inhibition); Swerdlow et al., 2008; San-Martin et al., 2020] but not in first-degree relatives of individuals with SCZ (review in Li et al., 2021).

A third variable, startle latency, has been measured in human 22qDel studies but not animal studies. Startle latency is the time between the startling stimulus and the startle response. Latency serves as a marker for speed of neural processing, and SCZ is associated with slower latency in several studies (Braff et al., 1978, 1999; Swerdlow et al., 2006; Hasenkamp et al., 2010; Smith et al., 2017; Massa et al., 2020) but not all (Braff et al., 1992; Parwani et al., 2000).

ASR PPI, magnitude, and latency are highly heritable, making them attractive investigation targets for research on the genetics of SCZ (Anokhin et al., 2003; Hasenkamp et al., 2010; Quednow et al., 2011; Greenwood et al., 2020; Massa et al., 2020).

The ASR is affected by many variables. Startle paradigm factors include background noise; the volume, duration, and rise-and-fall time of the startling tone; and whether a non-startling tone precedes the startling (Hoffman and Searle, 1968; Ison and Hoffman, 1983; Zheng and Schmid, 2023). Startle responses are also affected by exposure to the startling stimulus over time; responsivity can be decreased by habituation (Hoffman and Searle, 1968), where the subject acclimates to the stimulus, and increased by fear potentiation, where the startling tone is linked to an aversive stimulus (Davis et al., 1993; Grillon and Baas, 2003). In humans, ASR is affected by age, gender, menstrual cycle phase, medication status, genetic background, and smoking status (Swerdlow et al., 1997, 2006; Braff et al., 2001; Duncan et al., 2001; Ludewig et al., 2002; Jovanovic et al., 2004; Gebhardt et al., 2012; Fargotstein et al., 2018; Swerdlow and Light, 2018). In a meta-analysis, PPI was also related to education level and continent (potentially indicating a relationship between ethnicity and PPI) (San-Martin et al., 2020). Neurobiologically, startle magnitude is decreased by NMDA and non-NMDA glutamate antagonists (Miserendino and Davis, 1993) and increased by direct and indirect dopamine agonists (Kehne and Sorenson, 1978; Davis, 1980; Johansson et al., 1995; Martinez et al., 1999; Swerdlow et al., 2002).

Psychosis research has focused on several genes in the human 22q11.2 region. The catechol-O-methyl-transferase (*COMT*) gene and the proline dehydrogenase 1 (*PRODH*) gene mediate the ASR and potentially both mediate dopamine levels (Parker et al., 2024). The *COMT* gene encodes an enzyme critical to dopamine catabolism in the prefrontal cortex. The *COMT*-Val allele codes for a high-activity enzyme, and the *COMT*-Met allele codes for a low-activity enzyme (Paylor and Lindsay, 2006). *COMT*-Met associates with higher cortical dopamine and lower midbrain dopamine levels (Chen et al., 2004; Meyer-Lindenberg et al., 2005). In human 22qDel studies, PPI has been lower in *COMT*-Met than *COMT*-Val (Vorstman et al., 2009; De Koning et al., 2012). Another 22q11.2 region gene, *PRODH*, affects proline levels, and higher proline levels can increase dopamine release in the prefrontal cortex (Stark et al., 2009; Vorstman et al., 2009). The *COMT* and *PRODH* genes may have an interaction that indicates a homeostatic response to elevated dopaminergic signaling in the frontal cortex (Paterlini et al., 2005). De Koning et al. found that *COMT*-Met interacted with high proline levels in 22qDel to yield further reduced startle magnitude (De Koning et al., 2015). A recent mouse paper has found that a third gene in the region syntenic to human 22q11.2, *Tbx1*, may selectively affect acoustic sensorimotor gating (Hiramoto et al., 2025).

## ASR in 22qDel humans

Previous 22qDel research has examined ASR in children (Sobin et al., 2005a, 2005b; Vorstman et al., 2009) as well as in adolescents and adults (De Koning et al., 2012, 2015; McCabe et al., 2014; Parker et al., 2024).

There have been mixed findings on startle magnitude in 22qDel. Most studies have found no difference in startle magnitude between 22qDel and control groups (Sobin et al., 2005a, 2005b; Vorstman et al., 2009; De Koning et al., 2012; McCabe et al., 2014), but a more recent study found decreased startle magnitude in 22qDel subjects (Parker et al., 2024). This difference was further evidenced by the significantly higher number of 22qDel subjects classified as non-startlers (i.e., did not have a measurable blink response to at least four of the first six startling stimuli): while 25% of control subjects were non-startlers, 64.5% of 22qDel subjects were non-startlers. McCabe et al. (2014) also found a non-significant trend (*p* = 0.07) toward more no-response pulse trials in the 22qDel group compared to their control group, and Sobin et al. (2005b) removed three 22qDel participants from PPI analysis for being non-startlers (compared to no control participants). While de Koning et al. (2012, 2015) did not report between-group magnitude differences, they found lower startle magnitude and PPI in participants with the *COM*T-Met allele than the *COMT*-Val allele. The magnitude was further decreased in hyperprolinemic subjects (2015).

Findings on PPI in 22qDel have also been mixed, with studies reporting reduced PPI in 22qDel children (Sobin et al., 2005a, 2005b; Vorstman et al., 2009) but not adults (De Koning et al., 2012). Sobin et al. (2005a, 2005b) report that reduced PPI in both their cohorts was largely driven by decreased PPI in 22qDel boys vs. control boys; the PPI difference in 22qDel vs. control girls was much smaller. 22qDel PPI could not be meaningfully computed in the Parker et al. cohort (2024) due to the number of prepulse trials that elicited no startle response.

Startle latency has been studied less than magnitude or PPI in human studies. Sobin et al. (2005a, 2005b) found no difference in latency between children with and without 22qDel, while Parker et al. (2024) found that latency was prolonged in adolescents and adults with 22qDel.

These studies are summarized in Table 1. The inconsistency of 22qDel ASR findings could be from differences in participant age, paradigm design, or the relatively small sample sizes of studies. Additional research will need to determine whether differences in 22qDel ASR are marked from a young age or if they emerge in the psychosis risk window from late adolescence to early adulthood.

**Table 1:** Startle studies in 22qDel.

Study	Ages	*N* (22qDel)	*N* (Con)	22qDel vs control results*			Comments
				Magnitude	Latency	PPI	
Sobin et al. (2005a)	Children	21	25	ND	ND	decreased	PPI ↓ largely seen in 22qDel boys vs. control boys. %PPI correlated with higher (less efficient) executive attention index scores.
Sobin et al. (2005b)	Children	25	23	ND	ND	decreased	PPI ↓ largely seen in 22qDel boys vs. control boys.
Vorstman et al. (2009)	Children, including adolescents	40	33	NA	NA	decreased	Nonsignificant trend toward ↓ PPI in COMT(Met) vs. COMT(Val) 22qDel subgroups.
de Koning et al. (2012)	Adults	23	21	ND	NA	ND	PPI and magnitude ↓ in 22qDel COMT(Met) (*n* = 15) vs. COMT(Val) (*n* = 8) subgroups.
McCabe et al. (2014)	Adolescents and young adults	17	19	ND	NA	ND	Nonsignificant trend toward ↓ PPI in 22qDel vs. controls in active tasks. No difference in passive PPI tasks.
Parker et al. (2024)	Adolescents and adults	31	32	decreased	slowed	NA	could not assess PPI because low startle

*ND indicates no difference between groups; NA indicates not assessed.

## ASR in 22qDel mouse models

Mouse models are useful for 22qDel research because mouse genomic region 16qA13 (MMU16) is thought to be syntenic to the human 22q11.2 region (Paylor and Lindsay, 2006). Mouse models used to study 22qDel vary in the size and location of the MMU16 deletion as well as in mouse genetic background. There is one strain modeling the most prevalent 3.0 mb deletion in humans (Del(3.0 Mb) in Saito et al., 2020), five strains approximating the less-common 1.5mB deletion (1. Df(16)1/+ in Lindsay et al., 1999; 2. Lgdel/+ in Merscher et al., 2001; 3. Df(16)A+/− in Stark et al., 2009; 4. Df(h22q11)/+ in Didriksen et al., 2017; 5. Del(1.5 Mb)/+ in Saito et al., 2021), and one strain modeling a 1.4 Mb deletion on a section of 22q11 that is rarely deleted on its own in humans (Del(1.4 Mb) + in Saito et al., 2021). There are also mouse lines modeling deletions of smaller sections of the 22q11 region or with single gene knockouts from the deletion region (Kimber et al., 1999; Paylor et al., 2006). This review addresses mouse models with deletions covering a substantial part of the 16qA13 region. See Tables 2, 3 for information on the mouse lines reviewed. For more detailed information about 22qDel mouse models, see Ellegood et al., 2014; Meechan et al., 2015; Paylor and Lindsay, 2006; and Saito et al., 2020, 2021. For insight into the opportunities and limits of using mouse models in the context of schizophrenia, see Villarin and Kellendonk, 2025.

**Table 2:** Mouse 22qDel models,1.2mb + deletions.

Model name	First paper	Deletion region	Mouse background
Df1/+ (or Df(16)1/+)	Lindsay et al. (1999)	*Dgcr14-Ufd11*	Mixed C57/Bl6C-/C-; 129S5/SvEvBrd (noncongenic). backcrossed Df1/+ males w/ WT C57BL/6c−/c- females for 4–5 generations
Lgdel/+	Merscher et al. (2001)	*Dgcr2-Hira*	C57/B16N (noncongenic)
Df(16)A+/−	Stark et al. (2009)	*Dgcr2-Hira*	C57/B16J (noncongenic)
Df(h22q11)/+	Didriksen et al. (2017)	*Dgcr2-Hira*	C57/B16J (noncongenic)
Del(1.5 Mb)/+	Saito et al. (2021)	*Dgcr2-Hira*	backcrossed to C57BL/6 N for 3 generations
Del(1.4 Mb)/+	Saito et al. (2021)	*Pi4ka-Dgcr2*	backcrossed to C57BL/6 N for 3 generations
Del(3.0 Mb)/+	Saito et al. (2020)	*Pi4ka-Hira*	backcrossed to C57BL/6 N for 4 generations

**Table 3:** Mouse 22qDel models, multi-gene partial deletions.

Model name	First paper	Deletion region	Mouse background
unnamed	Kimber et al. (1999)	*Znf74-Ctp*	129SvEvTac or mixed 129SvEvTac (noncongenic)
Df2	Lindsay et al. (2001)	*Es2el-Txmd2*	N5–6 C57BL/6^c−/c−^ background. Backcrossed C57BL/6^c−/c−^;129S5/SvEvBrd mice with C57BL/6^c−/c−^ mice for 5–6 generations
Df3	Lindsay et al. (2001)	*Es2el-Cdcrel1*	see Df2
Df4	Lindsay et al. (2001)	*T10-Hira*	see Df2
Df5	Paylor et al. (2006)	*?-Hira*	see Df2

As shown in Tables 4, 5, PPI has been consistently reduced in 22qDel mouse models (Paylor et al., 2001, 2006; Long et al., 2006; Stark et al., 2009; Sigurdsson, 2016; Didriksen et al., 2017; Scarborough et al., 2019; Saito et al., 2020, 2021; Tripathi et al., 2020). The only model that has been tested for PPI that has not shown reduced PPI is Del(1.4 Mb)/+, which intentionally isolates a part of the deletion region that is not ever deleted in isolation in humans (Saito et al., 2021). Conversely, ASR magnitude has been increased in most mouse models [Del(3.0 Mb)/+; Df(h22q11)/+; LgDel; Df(16)1/+], though there was no difference found in two models [Del(1.4 Mb)/+, which does not replicate human 22qDel (Saito et al., 2021); and Df(16)A+/− (Stark et al., 2009)]. With the exception of a 150 kb deletion model (Kimber et al., 1999), partial models of the deletion have either shown increased magnitude and decreased latency or no difference (Paylor et al., 2006). Kimber et al.’s model demonstrated no difference in magnitude and increased PPI. Notably, no mouse data have been published on startle response latency in 22qDel.

**Table 4:** Startle in 22qDel mouse models (1.2mb+).

Study	Model name	Age	n (transgenic)	n (WT)	Transgenic vs WT Results*		
					Magnitude	Latency	PPI
Saito et al. (2021)	Del(1.5 Mb)/+	8–11 weeks	12 (all male)	12 (all male)	ND	NA	decreased
Saito et al. (2021)	Del(1.4 Mb)/+	8–11 weeks	12 (all male)	12 (all male)	ND	NA	ND
Saito et al. (2020)	Del(3.0 Mb)/+	2–5 months	15 (all male)	15 (all male)	increased	NA	decreased
Didriksen et al. (2017)	Df(h22q11)/+	6–21 weeks	91 (all male)	91 (all male)	increased	NA	decreased
Stark et al. (2009)	Df(16)A+/−	18 weeks	31 (21 m, 10 f)	29 (19 m, 10 f)	ND	NA	decreased
Long et al. (2006)	LgDel (Lgdel/+)	3–4 months	14 (6 m, 8 f)	12 (7 m, 5 f)	increased	NA	decreased
Paylor et al. (2001)	Df1/+ (Df(16)1/+)	8 weeks	19 (10 m, 9 f)	21 (11 m, 10 f)	increased	NA	decreased
Paylor et al. (2006)	Df1/+ (Df(16)1/+)	8–16 weeks	6 (? m, ? f)	6 (? m, ? f)	increased	NA	decreased
Scarborough et al. (2019)	Df(h22q11)/+	12–14 weeks	14 (all male)	11 (all male)	increased	NA	decreased
Tripathi et al. (2020)	Df(h22q11)/+	10–13 weeks	7–10 (all male)	7–10 (all male)	increased	NA	decreased

*ND indicates no difference between groups; NA indicates not assessed.

**Table 5:** Startle in 22qDel mouse models (partial deletions).

Study	Model name	Age	n (transgenic)	n (WT)	Transgenic vs WT Results*		
					Magnitude	Latency	PPI
Kimber et al. (1999)	unnamed	3–4 months	18 (all male)	14 (all male)	ND	NA	increased
Paylor et al. (2006)	Df2	8–16 weeks	*25–35 total case and WT littermates, m and f*		ND	NA	ND
Paylor et al. (2006)	Df3	8–16 weeks	*25–35 total case and WT littermates, m and f*		increased	NA	decreased
Paylor et al. (2006)	Df4	8–16 weeks	*25–35 total case and WT littermates, m and f*		increased	NA	decreased
Paylor et al. (2006)	Df5	8–16 weeks	*25–35 total case and WT littermates, m and f*		ND	NA	ND

*ND indicates no difference between groups; NA indicates not assessed.

## Discussion of 22qDel translational findings

Current ASR findings between mouse and human 22qDel research show similar overall trends, with greater internal consistency among mouse findings. PPI is reduced in all mouse models of 22qDel (Paylor et al., 2001, 2006; Long et al., 2006; Stark et al., 2009; Sigurdsson, 2016; Didriksen et al., 2017; Scarborough et al., 2019; Saito et al., 2020, 2021; Tripathi et al., 2020), and PPI in people with 22qDel has been either reduced (Sobin et al., 2005a, 2005b; Vorstman et al., 2009) or no significant PPI difference was found (De Koning et al., 2012; McCabe et al., 2014). Startle magnitude findings have been more divergent. Most human studies showed no difference in startle magnitude between 22qDel and control groups (reduced magnitude in 22qDel in Parker et al., 2024; no difference in Sobin et al., 2005a, 2005b; De Koning et al., 2012; McCabe et al., 2014). While two mouse studies found no magnitude difference in 22qDel models (Stark et al., 2009; Saito et al., 2021), most found a significant startle magnitude increase in 22qDel mice (Paylor et al., 2001, 2006; Long et al., 2006; Didriksen et al., 2017; Scarborough et al., 2019; Saito et al., 2020; Tripathi et al., 2020). To our knowledge, startle latency has not yet been studied in mice.

There are several factors that could inform the inconsistencies within human data and between human and mouse studies. First, the ASR is affected by estrogen. Menstrual cycle phase significantly affects PPI in adult humans (Swerdlow et al., 1997; Jovanovic et al., 2004), and estrogen levels modulate PPI and startle latency in mice (Charitidi et al., 2012). It is thought that the estrogen effect on PPI is mediated by estrogen effects on dopaminergic and possibly serotonergic signaling (see Koch, 1998; and discussions in Swerdlow et al., 1997; Jovanovic et al., 2004). Two human studies have found that differences in PPI in 22qDel have been largely driven by decreased PPI in 22qDel boys vs. control boys (Sobin et al., 2005a, 2005b). In contrast, another study found greater startle latency in CHR participants who later converted to psychosis, but only in females (Cadenhead et al., 2020). Only two out of 10 experiments on 22qDel model mice have included female mice; the greater consistency in mouse results, especially PPI, could potentially be due to more stable sample estrogen levels.

The human studies have varied in participant age, which complicates cross comparison. The human startle response changes significantly through childhood, and PPI matures around age 9–10 (Gebhardt et al., 2012). Adolescent PPI changes in healthy boys are more pronounced than in healthy girls (Giannopoulos et al., 2022). PPI also changes more slowly throughout the course of adulthood (Ellwanger et al., 2003). It is unknown to what extent ASR differences between individuals with and without 22qDel may be influenced by age. At this point, the only studies reporting decreased PPI in humans with 22qDel have been on children (Sobin et al., 2005a, 2005b; Vorstman et al., 2009). Mouse age has varied across studies, though most mice have been post-puberty (except possibly Didriksen et al., 2017). Hearing loss is a 22qDel phenotype that must be addressed in mouse and human ASR research. 22qDel mouse models generally have poor hearing (Fuchs et al., 2013), and they also display delayed-onset auditory thalamocortical projections that emerge between 2 and 4 months of age (Chun et al., 2014, 2017).

Mouse ASR is also influenced by genetic background (Pietropaolo and Crusio, 2009), and the genetic background of human 22qDel carriers affects their likelihood of developing psychiatric symptoms (Bassett et al., 2017; Cleynen et al., 2021). Obviously, genetic background varies widely in human 22qDel research. While several different genetic lines have been used for 22qDel mouse models, genetic background is far more controlled, which may also explain the consistency across mouse results. Finally, studies on both humans and mice have had relatively small sample sizes (see Tables 1, 4, 5).

It is noteworthy that the increased startle magnitude observed in 22qDel mouse models does not align with startle magnitude findings in the context of psychosis. While some studies on startle magnitude in psychosis have found reduced magnitude in SCZ vs. control participants (Kumari et al., 2005; Quednow et al., 2006; Minassian et al., 2007; Matsuo et al., 2016; Greenwood et al., 2020), most have found no significant difference between groups (Braff et al., 1992, 2005; Cadenhead et al., 2000; Parwani et al., 2000; Ludewig et al., 2002; Perry et al., 2002; Wynn et al., 2004; Swerdlow et al., 2006; Takahashi et al., 2008; Hasenkamp et al., 2010; Storozheva et al., 2016). This is consistent with human 22qDel research findings, but inconsistent with the reliably increased startle magnitude in 22qDel mice models. On the other hand, the decrease in PPI found in all mouse models of 22qDel and in three of the five human 22qDel studies on PPI is consistent with PPI reduction as a SCZ endophenotype (as reviewed in Swerdlow and Light, 2018).

Finally, it is important to acknowledge the role of medication as a confounding factor in research on 22qDel patients. Because 22qDel patients are at elevated risk for a variety of psychiatric syndromes, including ADHD, they are more likely to be prescribed a variety of psychotropic medications (Fiksinski et al., 2021). This includes antipsychotics which tend to normalize startle parameters, and psychostimulants which may amplify them (Ashare et al., 2010). Protocols for research with clinical samples typically do not include restriction of prescribed medications, thus we are not aware of any published studies of 22qDel samples that include only nonmedicated patients. Further, subgrouping 22q Del patients based on medication status diminishes statistical power for detecting differences from healthy controls. While this limitation is unavoidable in human studies, it is not relevant to animal models, and likely explains the greater consistency in studies of ASR based on animal models of 22qDel.

## Conclusion

Translational ASR research has advanced our understanding of the neurobiology underlying several psychiatric disorders (Davis et al., 1993; Braff et al., 2001; Swerdlow et al., 2001, 2016). The ASR can be easily studied across species using analogous paradigms. Since startle circuitry and neurochemistry have been established in animal models (Lee et al., 1996; Koch, 1999; Fendt et al., 2001; Zheng and Schmid, 2023), ASR abnormalities in human conditions can provide insight into the neurobiology of those conditions. 22qDel research matters in its own right, and, given the high risk of SCZ development that 22qDel confers, this research will provide important inroads into the neurobiology of psychosis. To date, there are areas of convergence and divergence in ASR variables between human and mouse 22qDel studies. Further translational study of the ASR in 22qDel holds promise for identifying underlying neurobiology and potential treatment targets for 22qDel and SCZ.
